# Environmental Enrichment Elicits a Transient Rise of Bioactive C-Type Natriuretic Peptide in Young but Not Aged Rats

**DOI:** 10.3389/fnbeh.2018.00142

**Published:** 2018-07-19

**Authors:** Susan A. Rapley, Timothy C. R. Prickett, John C. Dalrymple-Alford, Eric A. Espiner

**Affiliations:** ^1^Brain Research New Zealand and Psychology, University of Canterbury, Christchurch, New Zealand; ^2^Department of Medicine, University of Otago, Christchurch, New Zealand

**Keywords:** C-type natriuretic peptide, environmental enrichment, aging, medial prefrontal cortex, hippocampus, retrosplenial cortex

## Abstract

Beneficial molecular and neuroplastic changes have been demonstrated in response to environmental enrichment (EE) in laboratory animals across the lifespan. Here, we investigated whether these effects extend to C-type Natriuretic Peptide (CNP), a widely expressed neuropeptide with putative involvement in neuroprotection, neuroplasticity, anxiety, and learning and memory. We determined the CNP response in 36 young (8–9 months) and 36 aged (22–23 months) male PVGc hooded rats that were rehoused with new cage mates in either standard laboratory cages or EE for periods of 14 or 28 days. Tissues were rapidly excised from four brain regions associated with memory formation (dorsal hippocampus, retrosplenial cortex, medial prefrontal cortex, and mammillary bodies) plus the occipital cortex and hypothalamus, and immediately frozen. Radioimmunoassay was used to measure bioactive CNP and the amino-terminal fragment of proCNP, NTproCNP. Because CNP but not NTproCNP is rapidly degraded at source, NTproCNP reflects CNP production whereas the ratio NTproCNP:CNP is a biomarker of CNP's local degradation rate. EE increased CNP at 14 days in all brain regions in young, but not old rats; this effect in young rats was lost at 28 days in all regions of interest. NTproCNP:CNP ratio, but not NTproCNP, was reduced in all regions by EE at 14 days in young rats, but not in old rats, which suggests a period of reduced degradation or receptor mediated clearance, rather than increased production of CNP in these young EE rats. Aged rats tended to show reduced NTproCNP:CNP ratios but this did not occur in dorsal hippocampus or mammillary bodies. This is the first study demonstrating modulation of CNP protein concentrations, and the effect of age, in response to environmental stimulation. Furthermore, it is the first to show that changes in degradation rate *in vivo* may be an important component in determining CNP bioactivity in neural tissues.

## Introduction

Multiple beneficial effects of Environmental Enrichment (EE) have been established over 60 years of research using the paradigm. These variously occur at molecular, cellular and behavioral levels and relate to neurogenesis, synaptic plasticity, neuroprotection, and modifications to anxiety and learning and memory across the lifespan (see Rosenzweig and Bennett, 1996; van Praag et al., 2000; Will et al., 2004; Nithianantharajah and Hannan, 2006; Clemenson et al., 2015 for selected reviews). Less well-established is the neurological action of C-type Natriuretic Peptide (CNP), a proposed neuroendocrine regulator, with similar associations to those effects modified by EE.

CNP is the most recently discovered, but most primitive member of the Natriuretic Peptide family alongside Atrial and B-type Natriuretic Peptides. The family shares a ring structure consisting of 17 amino acid residues, flanked by two cysteine residues forming a disulfide bond critical to biological activity (Pandey, 2005; Potter et al., 2006). Three forms of the peptide occur: expression of the Nppc (Natriuretic Peptide Precursor-C) gene elicits the initial 126 residue preproCNP (Tawaragi et al., 1990) resulting in the 103 residue proCNP, which is cleaved intracellularly to produce CNP-53 and a biologically inactive amino terminal fragment, NTproCNP, both of which are secreted extracellularly in equimolar amounts (Wu et al., 2003; Prickett and Espiner, 2012). Further extracellular processing results in CNP-22, thought to be the fully active form of the peptide, with CNP-53 proposed as a storage form (Barr et al., 1996). CNP-22 is the most highly conserved of the Natriuretic Peptide family and is identical in all mammals studied to date (Pandey, 2005; Potter, 2011). CNP binds to a specific receptor, Natriuretic Peptide Receptor-B (NPR-B), through which it increases intracellular concentrations of cyclic Guanosine Monophosphate (cGMP; Yeung et al., 1996; Potter et al., 2006; Potter, 2011). Regulation of the peptide occurs via a membrane bound clearance receptor (NPR-C), which clears all Natriuretic Peptides from the extracellular space; and proteolytic degradation by neprilysin (primarily) and insulin degrading enzyme (Müller et al., 1992; Kenny et al., 1993; Watanabe et al., 1997; Potter, 2011).

It is important to note that the concentration of CNP at any time point is a function of both production and clearance or degradation rates. Since NTproCNP is not subject to rapid degradation or clearance (half-life of 30–40 min compared with 2–3 min for CNP), it provides a reliable measure of NPPC gene expression and peptide production (Schouten et al., 2011; Woodward et al., 2017). Calculation of NTproCNP:CNP ratio (by taking NTproCNP/CNP concentrations) provides an index of CNP's clearance or degradation in the tissue sampled, either via NPR-C mediated internalization or hydrolysis by neprilysin. Higher ratios reflect increases in the rate of loss of the bioactive form of the peptide. Combined measurement of CNP and NTproCNP, with calculation of the ratio, allows separate contributions of peptide production and metabolic clearance to be determined.

High concentrations of CNP are found in brain tissue, with the peptide constituting the major neuroactive member of the natriuretic peptide family (Komatsu et al., 1991; Kaneko et al., 1993; Totsune et al., 1994; Langub et al., 1995a; Pemberton et al., 2002; Jankowski et al., 2004; Wilson et al., 2017). In rodents, both CNP and NPR-B mRNA occur variably throughout olfactory bulb, basal forebrain, basal ganglia, limbic cortices, thalamus, amygdala, mammillary nuclei, hippocampus, and cerebellum (Langub et al., 1995a,b; Herman et al., 1996). As CNP is widely accepted to act in a paracrine/autocrine fashion, this regional distribution suggests a role for the peptide in the integration of sensation and emotion into memory.

This hypothesis receives initial support from the relatively sparse literature regarding CNP's effects within brain, and on behavior. Additionally, these findings indicate similarities between CNP's action and the beneficial effects of EE specifically in terms of neurogenesis, neuroplasticity, modification of anxiety behaviors, learning and memory. CNP induces a switch from proliferation to maturation in olfactory neuronal precursors (Simpson et al., 2002), is necessary for bifurcation of sensory axons (Schmidt et al., 2007, 2009; Zhao and Ma, 2009) and is associated with the onset of neurogenesis within the developing nervous system (DiCicco-Bloom et al., 2004). Within retinal ganglion cells, CNP is neuroprotective against cytotoxic injury both *in vivo* and *in vitro* (Ma et al., 2010). Within the hippocampus, CNP modifies several electrophysiological correlates of synaptic plasticity, and notably impedes long-term potentiation while facilitating long-term depression within CA1 (Decker et al., 2008, 2009, 2010). In relation to anxiety behavior, CNP may have a bidirectional effect as it is anxiolytic in low dose ranges, but anxiogenic when higher doses are administered (Bíró et al., 1996; Montkowski et al., 1998; Jahn et al., 2001). Such a bidirectional effect may also occur in relation to learning and memory behaviors: CNP improves learning in a passive avoidance task (Telegdy et al., 1999; Telegdy et al., 2000), but functional downregulation of NPR-B receptor signaling is associated with improved object-recognition memory (Barmashenko et al., 2014). Based on these emerging similarities between CNP's effects and effects demonstrated in EE, we posited that the paradigm would provide an excellent context for study of CNP within brain tissue.

In addition to a possible effect of EE on CNP, it has been recently suggested that Natriuretic Peptides may be involved in cognitive impairment and pathologies related to aging (Mahinrad et al., 2016). Although no relationship was previously found between age and CNP concentration in cerebrospinal fluid across a specific age-range in Parkinson's Disease patients (51–90 years; Schouten et al., 2011; Espiner et al., 2014), this does not exclude the possibility that regional variations may still be found within cerebral tissue. A primary question is whether CNP expression or degradation within brain tissue varies during normal aging. To this end, we analyzed brain tissue of both young and aged rats housed in either standard or enriched cages for two different periods of housing, selected to be representative of shorter- and longer-term exposure typical of many enrichment studies (Simpson and Kelly, 2011). Given previously demonstrated reductions of neprilysin in fronto-temporal regions with brain aging (Iwata et al., 2002; Apelt et al., 2003; Fjell et al., 2014), general reductions to the NTproCNP:CNP ratio were expected in aged rats. As this is the first study of its kind, no predictions could be made about how enriched housing may affect concentrations of CNP, or synthesis of the peptide measured by NTproCNP concentrations in either age group.

## Methods

### Subjects

Thirty-six male PVGc hooded rats aged 8–9 months old (weights between 306 and 413 g) and 36 male PVGc hooded rats aged 22–23 months (weights between 293 and 440 g) at the start of enrichment were treated equivalently in all respects. Prior to enrichment, all rats were housed in standard opaque plastic cages (45 × 27 × 22 cm high) in groups of three or four, from weaning until placement in enrichment at the stated ages. For each age group, on the first day of enrichment, 24 rats were re-housed in two enrichment cages (12 per cage) for either 14 or 28 days (1 cage per time-period; Enriched-14-day and Enriched-28-day). Twelve remaining rats were re-housed with new cage mates in standard cages (3 rats per cage) for the same time periods (2 cages per time-period; 6 rats total per time-period; Standard-14-day and Standard-28-day). All rats were rehoused with novel cage mates. Rats were maintained on a reversed light-dark cycle (lights off 08:00–20:00 h) and colony rooms were maintained at 22°C and 48% relative humidity. Food and water were available *ad libitum*. All procedures conformed to the NIH guide for the care and use of laboratory animals and were approved by the University of Canterbury Animal Ethics Committee.

### Enrichment

A standardized enrichment protocol developed at the University of Canterbury was used (Harland et al., 2014; and see http://www.psyc.canterbury.ac.nz/Standardized%20Enrichment.shtml for details of, objects, arrangements and procedures). Enrichment cages were made of wire mesh with a sawdust covered metal floor and measured 85 cm x 60 cm x 30 cm high. Each day of enrichment consisted of a different combination of “junk” objects such as ceramic figurines, metal chains, PVC pipes and junctions and other small items, along with an ever-present wooden block to discourage chewing of enrichment objects. Enrichment configurations differed over 40 different days (though a maximum of 28 was used here) and ensured no object was repeated within 5 days of itself. On every seventh day, PVC pipes and junctions were presented in a “tubing only” day, and on every eighth day, all objects (except the wooden blocks) were removed from the cage. Additionally, food and water positions were changed daily, and cages were rotated through one of four possible positions in the colony room every fourth day. Objects were changed at the start of the dark period (between 09:00 and10:00h), during which rats from one enrichment cage were held together in a large opaque plastic cage (62 × 40 × 22 cm high).

### Sacrifice and tissue extraction

After either 14 or 28 full days of enrichment, or the same period of standard housing with new cage mates, rats were placed in standard cages at the same time enrichment objects were usually changed and held in a novel, dimly lit room separate to both the colony room and tissue dissection room. Rats from Enriched and Standard Housing were euthanised throughout a single day, with two Enriched rats sacrificed per one Standard Housed, and order of sacrifice randomized within these groups of three. Rats were deeply anesthetised with an overdose of Sodium Pentobarbitone (1mL, 300 mg/mL, ip). Once unresponsive to both tail pinch and plantar reflex (~5 min following injection), with no discernible heartbeat, rats were decapitated, and brains rapidly removed from the skull and placed in a brain matrix (Ted Pella). An initial coronal cut was made at the level of the optic tract. Additional coronal cuts were made 5 mm anterior and posterior to the initial cut, resulting in two “slabs” of fresh tissue (anterior extent approximately equivalent to Bregma +3.20 mm; posterior extent approximately equivalent to Bregma −6.30 mm; from the atlas of Paxinos and Watson, 1998). These tissue slabs were placed anterior-face-upwards on a glass petri dish, previously sterilized with 70% ethanol and rinsed with saline. Sterilization and rinsing was repeated between rats. Microdissection scissors were used to acquire tissue samples from seven regions of interest, starting in the posterior slab, in the order: occipital cortex, retrosplenial cortex (containing both dysgranular and granular B tissue), dorsal hippocampus (left and right hemispheres separately), mammillary bodies, hypothalamus and medial prefrontal cortex. Tissue samples were placed in pre-weighed Eppendorf tubes, weighed, and snap frozen with liquid nitrogen, within 15 min post-sacrifice, before long-term storage at −80°C.

### Tissue preparation and peptide measurements

CNP and NTproCNP were measured by radioimmunoassay (RIA), described in detail in (Yandle et al. (1993) CNP) and (Yandle et al. (1993) NTproCNP). Prior to assay, frozen tissue samples were transferred to dry ice chilled scintillation vials, then 10 mL of boiling distilled water containing 0.01% Triton X-100 was added and the vials were held at 98°C in a water bath for 5 min. Samples were acidified with 610 μL glacial acetic acid and homogenized prior to extraction on Sep-Pak C18 cartridges (Waters Corporation, Milford, MA, USA). Following extraction, samples were dried under an air stream and frozen for later re-suspension in assay buffer for RIA. All tissues from an individual rat were processed in the same extraction, with tissues from housing groups counterbalanced across extractions.

#### CNP assay

CNP-22 is identical in all mammals studied thus far. CNP-22 and the amino terminal extended form CNP-53, both contain the 17-amino acid ring essential for bioactivity and show 100% cross reactivity in the assay used to measure CNP. Antiserum to CNP-22 (G-012-03, Phoenix Pharmaceuticals, Belmont, CA) was diluted 1:2,000 with assay buffer. Labeled CNP was prepared by chloramine-T iodination of [Tyr0] CNP-22 (Peninsula Labs) and purified by reverse-phase HPLC. Fifty microliters each of antiserum and CNP standard (0.7–235 pmol/L) or sample extract (all in duplicate) were mixed and incubated for 22–24 h at 4°C, followed by addition of 50 μL labeled CNP containing 2,000 cpm for 22–24 h at 4°C. Bound and free labeled CNP were separated by a solid-phase secondary antibody method (Sac-cell, Rabbit-Anti Goat, IDS Ltd., England). CNP assays had a detection limit of 0.6 pmol/L and ED50 of 7.3 pmol/L; intra- and inter-assay coefficients of variation were 5.9 and 7.4%, respectively, at 17 pmol/L.

#### NTproCNP assay

For NTproCNP an in-house antiserum was used that recognizes the C-terminal epitope in the region of proCNP (38–50), which is identical in human, mouse, and rat (Prickett et al., 2012). Fifty microliters of sample extract or 0.5–372 pmol/L proCNP(36–50) standards (again in duplicate) were incubated with 50 μL ovine antiserum (Sheep43) for 22–24 h, followed by addition of 50 μL tracer solution (proCNP(38-50)-[125I]Tyr37) containing 2,000 cpm for 22–24 h at 4°C. Bound and free labeled proCNP were separated by solid-phase second antibody method (Sac-cell, Donkey-Anti Sheep, IDS Ltd., England). NTproCNP assays had a detection limit of 0.4 pmol/L and ED50 9.9 pmol/L; intra- and inter-assay coefficients of variation were 6.8 and 7.5%, respectively, at 45 pmol/L.

### Statistical analyses

CNP and NTproCNP concentrations were expressed as femtomoles per gram of wet tissue (fmol/g). NTproCNP:CNP ratio was calculated by dividing NTproCNP concentration by CNP concentration. In young rats, CNP assay failed in one sample from each of retrosplenial (Standard-14-day group) and occipital (Enriched-14-day group) cortices and data was excluded, reducing sample Ns in these regions. In aged rats, a portion of one sample from the hypothalamus (Enriched-28-day group) was lost, and data subsequently excluded. Two tissue samples had NTproCNP concentrations beyond the detection limit of the assay: one from mammillary bodies (Enriched-14-day group) and one from hypothalamus (Enriched-28-day group). Thus, sample Ns for aged rats are reduced for each of these regions (mammillary bodies and hypothalamus). In both age groups, final Ns for all other regions were: Enriched-14-day, *N* = 12; Enriched-28-day, *N* = 12; Standard-14-day, *N* = 6; Standard-28-day, *N* = 6. Initial analysis indicated there were no differences in any measure (CNP, NTproCNP or ratio) between left and right hemispheres of the dorsal hippocampus. Data was averaged across hemispheres for each rat and analyzed as a single value. Data acquisition and analysis for Young rats was conducted prior to acquisition of Aged rat data. As the Null Hypothesis was rejected in most cases for Young rats (see results), data from Aged rats were compared directly using cohen's *d* effect sizes. Analysis of data for Young rats was by Robust 2-way ANOVA with 20% trimmed means (after Mair and Wilcox, 2016; using WRS2 package for “R”). The statistical outcome measure of this test—Q—is interpreted in the same fashion as the traditional F statistic. *Post-hoc* testing of Young rats and Aged rat comparisons by effect size (cohen's *d* [± 95% CI]) calculations were all based on trimmed measures (using compute.es package for “R”). Figures provide boxplots displaying trimmed mean, standard error and 95% confidence intervals, overlaid with all data points (constructed using ggplot2 for “R”).

## Results

### NTproCNP concentrations

**Figure 1:**
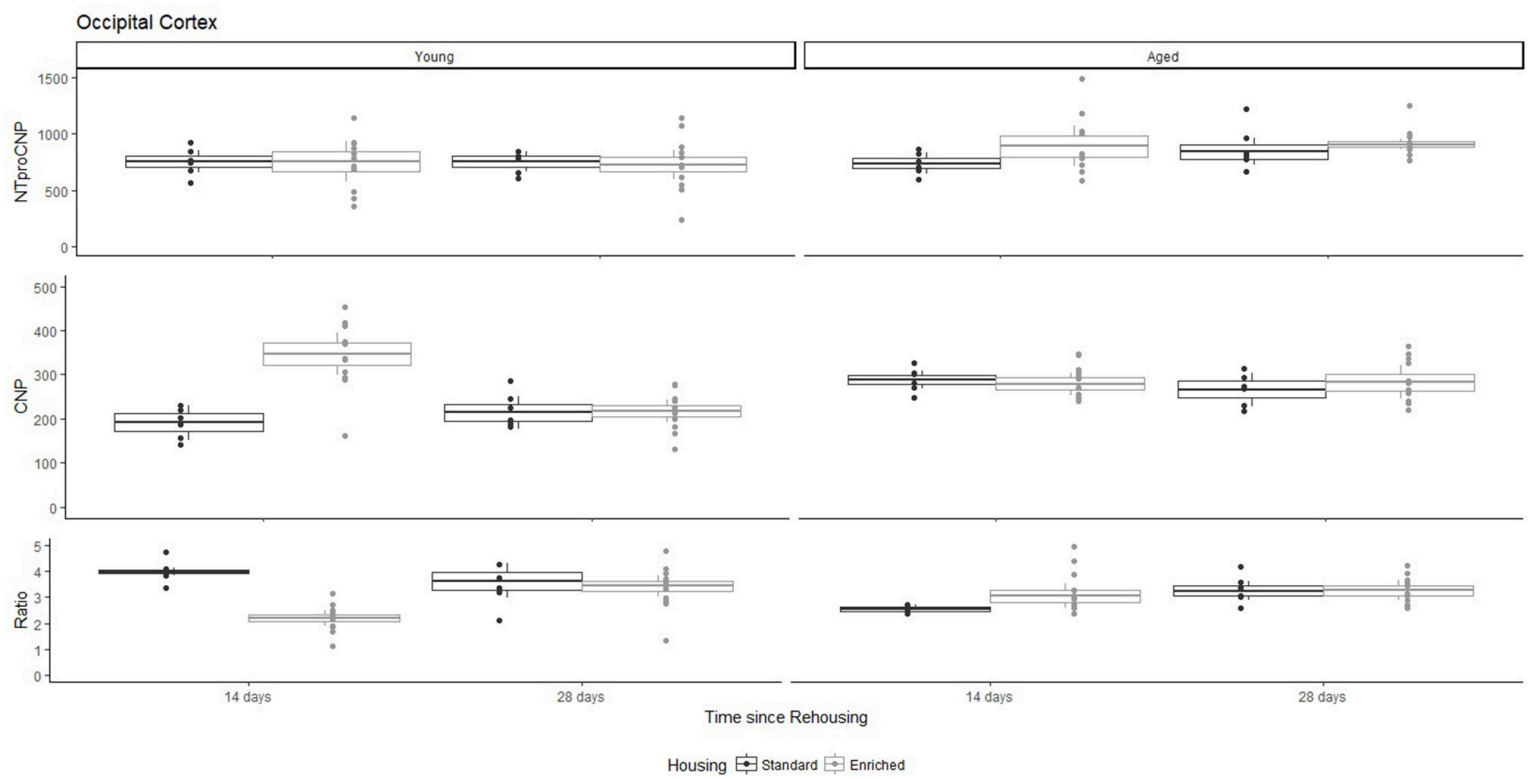
Concentration (femtomoles/g wet tissue) of NTproCNP **(Top)**, CNP **(Middle)**, and NTproCNP:CNP ratio **(Bottom)** within occipital cortex. Summary data is 20% trimmed means (resulting in a reduction of *n* = 2 for all groups), standard error (box) and 95% confidence interval (whisker), overlaid by individual data points. For NTproCNP: Aged rats > Young rats. For CNP: Young-Enriched-14-day > Aged overall > Other Young rats. For Ratio: Young-Enriched-14-day < Aged overall < Other Young rats.

**Figure 2:**
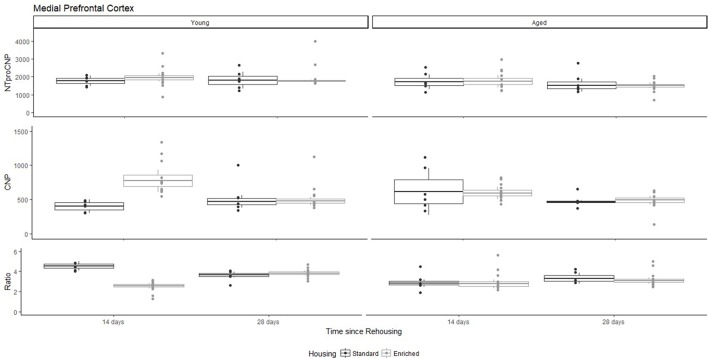
Concentration (femtomoles/g wet tissue) of NTproCNP **(Top)**, CNP **(Middle)**, and NTproCNP:CNP ratio **(Bottom)** within medial prefrontal cortex. Summary data is 20% trimmed means (resulting in a reduction of *n* = 2 for all groups), standard error (box) and 95% confidence interval (whisker), overlaid by individual data points. For NTproCNP: Aged rats < Young rats. For CNP: Young-Enriched-14-day > Other Young rats; Aged-14-day > Aged-28-day. For Ratio: Young-Enriched-14-day = Aged overall < Other Young rats.

**Figure 3:**
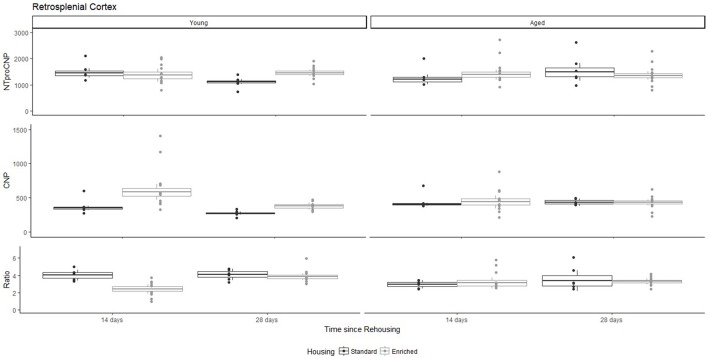
Concentration (femtomoles/g wet tissue) of NTproCNP **(Top)**, CNP **(Middle)**, and NTproCNP:CNP ratio **(Bottom)** within retrosplenial cortex. Summary data is 20% trimmed means (resulting in a reduction of *n* = 2 for all groups), standard error (box) and 95% confidence interval (whisker), overlaid by individual data points. For NTproCNP: Young-Standard-14-day > Young-Standard-28-day. For CNP: Young-Enriched-14-day > Aged overall > Other Young rats. For Ratio: Young-Enriched-14-day < Aged overall < Other Young rats.

**Figure 4:**
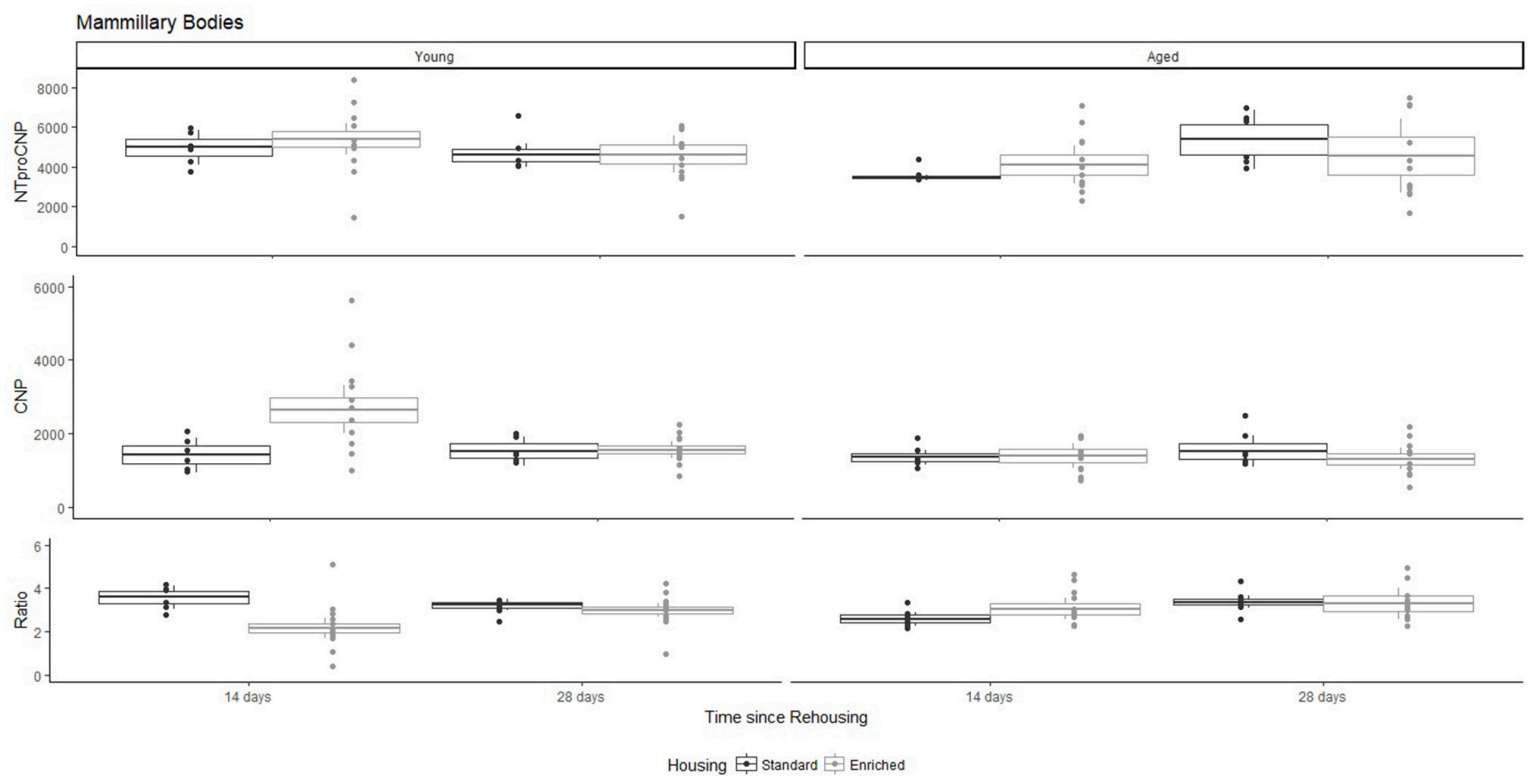
Concentration (femtomoles/g wet tissue) of NTproCNP **(Top)**, CNP **(Middle)**, and NTproCNP:CNP ratio **(Bottom)** within mammillary bodies. Summary data is 20% trimmed means (resulting in a reduction of *n* = 2 for all groups), standard error (box) and 95% confidence interval (whisker), overlaid by individual data points. For NTproCNP: Aged rats = Young rats. For CNP: Young-Enriched-14-day > Aged overall = Other Young rats. For Ratio: Young-Enriched-14-day < Aged overall = Other Young rats.

**Figure 5:**
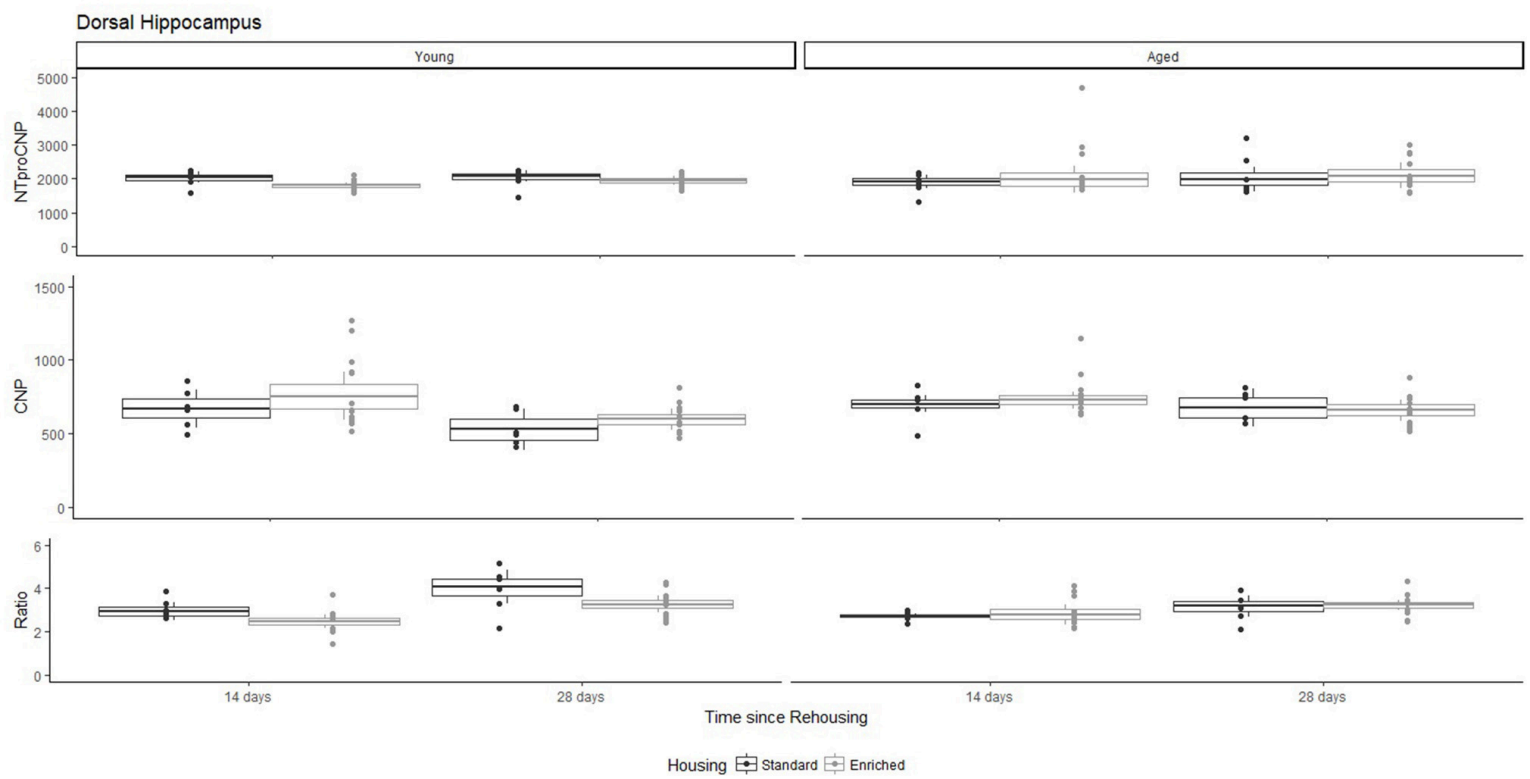
Concentration (femtomoles/g wet tissue) of NTproCNP **(Top)**, CNP **(Middle)**, and NTproCNP:CNP ratio **(Bottom)** within dorsal hippocampus. Summary data is 20% trimmed means (resulting in a reduction of n = 2 for all groups), standard error (box) and 95% confidence interval (whisker), overlaid by individual data points. For NTproCNP: Young-Enriched < Young-Standard. For CNP: Young-14-day = Aged overall > Young-28-day. For Ratio: Young-Enriched-14-day < Aged overall = Other Young rats; Young-14-day < Young-28-day.

**Figure 6:**
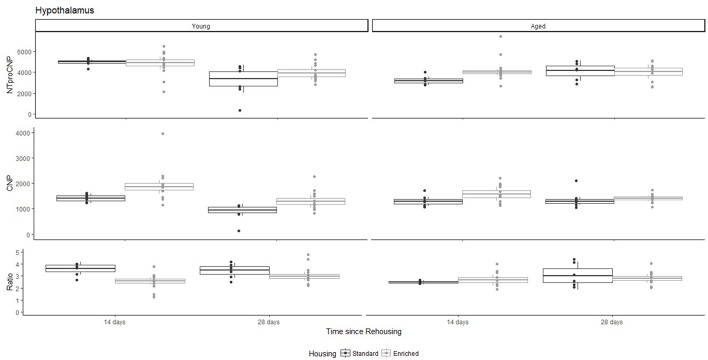
Concentration (femtomoles/g wet tissue) of NTproCNP **(Top)**, CNP **(Middle)**, and NTproCNP:CNP ratio **(Bottom)** within hypothalamus. Summary data is 20% trimmed means (resulting in a reduction of *n* = 2 for all groups), standard error (box) and 95% confidence interval (whisker), overlaid by individual data points. For NTproCNP: Young-14-day > Young-28-day. For CNP: Young-Enriched-14-day > Aged overall = Other Young rats. For Ratio: Young-Enriched-14-day < Young-Standard. Aged overall < Other Young rats.

For Young rats, NTproCNP concentrations did not vary in occipital cortex (Figure 1; overall M [95% CI] = 749.38 [691.36, 807.40]), medial prefrontal cortex (Figure 2; overall M [95% CI] = 1847.26 [1739.99, 1954.53]), or mammillary bodies (Figure 4; overall M [95% CI] = 4938.76 [4519.69, 5357.83]). NTproCNP concentrations also did not vary for Aged rats in occipital cortex (Figure 1; overall M [95% CI] = 854.61 [801.54, 907.68]), medial prefrontal cortex (Figure 2; overall M [95% CI] = 1599.42 [1448.72, 1750.12]), or mammillary bodies (Figure 4; overall M [95% CI] = 4245.68 [3547.89, 4943.48]). Concentrations of NTproCNP for Aged rats were higher than for Young rats in occipital cortex (Figure 1; *d* = 0.62 [0.14, 1.11], *p* = 0.01), lower than Young rats in medial prefrontal cortex (Figure 2; *d* = 0.62 [0.14, 1.10], *p* = 0.01), and equivalent in mammillary bodies (Figure 4). In retrosplenial cortex of Young rats (Figure 3), NTproCNP concentrations were lower at 14 days after rehousing than 28 days after rehousing, in Standard cages only (Housing x Time Interaction: *Q*_(1, 31)_ = 6.40, *p* = 0.026; Young-Standard-14-day vs. Young-Standard-28-day (*d* = 2.12 [0.41, 3.83], *p* = 0.02). No significant between group differences were identified for NTproCNP for Aged rats in retrosplenial cortex (Figure 3). In dorsal hippocampus, NTproCNP concentrations were lower in Enriched housing than Standard housing, regardless of Time since rehousing (Figure 5; Main effect of Housing: *Q*_(1, 32)_ = 7.55, *p* = 0.017; Young-Enriched vs. Young-Standard *d* = 0.87 [0.12, 1.62], *p* = 0.02). Again, no effects were identified in Aged rats (Figure 5). In hypothalamus, NTproCNP concentrations were higher following 14 days of rehousing than 28 days, regardless of Housing condition (Figure 6; Main effect of Time *Q*_(1, 32)_ = 11.05, *p* = 0.01; Young-14-day vs. Young-28-day *d* = 1.13 [0.4, 1.86], *p* < 0.001). This effect was also not apparent in Aged rats (Figure 6).

### CNP concentrations

For Young rats, significant two-way interactions (Housing × Time) were identified in occipital cortex [*Q*_(1, 31)_ = 16.77, *p* = 0.001], medial prefrontal cortex [*Q*_(1, 32)_ = 12.56, *p* = 0.003], retrosplenial cortex [*Q*_(1, 31)_ = 4.45, *p* = 0.055] and mammillary bodies [*Q*_(1, 32)_ = 7.41, *p* = 0.015]. In each region (Figures 1–4), CNP concentrations were elevated in Young-Enriched-14-day rats compared to all other groups of rats (between group comparisons, Table 1). Additionally, in retrosplenial cortex CNP concentrations were reduced across time since rehousing for Standard Housed rats (Young-Standard-14-days vs. Young-Standard-28-days *d* = 1.37 [0.21, 2.54], *p* = 0.02). In dorsal hippocampus, higher concentrations of CNP were seen 14 days after rehousing with new cage mates, regardless of type of Housing [Figure 5; Main Effect of Time: *Q*_(1, 32)_ = 5.74, *p* = 0.03; Young-14-day vs. Young-28-day *d* = 0.7 [0, 1.4], *p* = 0.05]. Although there was no significant effect of Housing, highest concentrations of CNP occurred within Young-Enriched-14-day rats (Figure 5). In hypothalamus, significant Main Effects of Housing [*Q*_(1, 32)_ = 12.66, *p* = 0.002] and Time [*Q*_(1, 32)_ = 21.00, *p* = 0.001] were such that CNP concentrations were higher in Enriched rats than Standard Housed rats, and higher 14 days following rehousing with new cage mates (Figure 6). The additive nature of these effects was reflected in significantly higher concentrations of CNP in Young-Enriched-14-day rats vs. all other groups (between group comparisons, Table 1).

**Table 1:** Between group comparisons (cohen's *d* [±95% CI]) for CNP concentrations within five regions of interest (see text for hippocampus and Aged medial prefrontal cortex).

**Region**		**Young SH 14**	**Young SH 28**	**Young EE 28**	**Aged overall**
Occipital Cortex	Young EE 14 vs.	*d* = 2.14 [0.8, 3.47] ^***^	*d* = 1.85 [0.57, 3.13]^**^	*d* = 1.98 [0.92, 3.04]^***^	*d* = 1.22 [0.48, 1.96]^***^
	Other Young vs.				*d* = −1.57 [−2.17, −0.97]^***^
Medial Prefrontal Cortex	Young EE 14 vs.	*d* = 1.50 [0.31, 2.68]^*^	*d* = 1.22 [0.08, 2.36]^*^	*d* = 1.37 [0.43, 2.31]^**^	
Retrosplenial Cortex	Young EE 14 vs.	*d* = 1.35 [0.11, 2.58]^*^	*d* = 1.84 [0.6, 3.09]^**^	*d* = 1.32 [0.38, 2.25]^**^	*d* = 1.31 [0.59, 2.03]^***^
	Other Young vs.				*d* = −1.13 [−1.71, −0.56]^***^
Mammillary Bodies	Young EE 14 vs.	*d* = 1.22 [0.08, 2.36]^*^	*d* = 1.13 [0.0, 2.26]^*^	*d* = 1.27 [0.34, 2.19]^**^	*d* = 1.75 [0.99, 2.52]^***^
	Other Young vs.				*d* = 0.30 [−0.24, 0.83] ns
Hypothalamus	Young EE 14 vs.	*d* = 1.11 [0.02, 2.24]^*^	*d* = 2.17 [0.86, 3.48]^***^	*d* = 1.26 [0.33, 2.18]^**^	*d* = 1.21 [0.49, 1.94]^***^
	Other Young vs.				*d* = −0.45 [−0.99, 0.09] ns

*EE, Enriched Environment; SH, Standard Housed; Other Young indicates pooled Young data, excluding Young-Enriched-14-day rats*.

*** *p < 0.001*;

** *p < 0.01*;

* *p < 0.05*.

For Aged rats, CNP concentrations did not vary significantly in any region, in individual group comparisons (Figures 1–6). The sole effect on CNP concentrations identified within Aged rats was in medial prefrontal cortex (Figure 2), wherein CNP concentrations were higher 14 days after rehousing with new cage mates than at 28 days of rehousing (*d* = 0.68 [−0.01, 1.38], *p* = 0.005). CNP concentrations for Aged rats overall were lower than the peak seen in Young-Enriched-14-day rats in occipital cortex, retrosplenial cortex, mammillary bodies and hypothalamus (between group comparisons, Table 1). In occipital and retrosplenial cortices (Figures 1, 3), CNP concentrations were higher in Aged rats than for other Young rats (i.e., excluding Young-Enriched-14-day rats; between group comparisons, Table 1). In the Aged hippocampus, concentrations of CNP were equivalent to Young rats rehoused for 14 days (*d* = 0.12 [−0.46, 0.47], ns), but higher than Young rats rehoused for 28 days (*d* = −1.01 [−1.62, −0.4], *p* < 0.001).

### NTproCNP:CNP ratio

For Young rats, significant two-way interactions were identified in occipital cortex [*Q*_(1, 31)_ = 14.76, *p* = 0.004], medial prefrontal cortex [*Q*_(1, 32)_ = 49.00, *p* = 0.001], retrosplenial cortex [*Q*_(1, 31)_ = 5.55, *p* = 0.039] and mammillary bodies [*Q*_(1, 32)_ = 9.20, *p* = 0.009]. In each region (Figures 1–4) NTproCNP:CNP ratio was reduced in Young-Enriched-14-day rats compared with all other Young rats (between group comparisons, Table 2). In dorsal hippocampus (Figure 5), NTproCNP:CNP ratio was lower 14 days following rehousing than at 28 days of rehousing [Main Effect of Time: *Q*_(1, 32)_ = 15.87, *p* = 0.003] and lower in rats housed in Enrichment than in Standard cages [Main Effect of Housing: *Q*_(1, 32)_ = 7.03, *p* = 0.026]. Lowest ratio values therefore occurred in Young-Enriched-14-day rats, but this difference was not significant compared with Young-Standard-14-day rats (between group comparisons, Table 2). In hypothalamus (Figure 6), NTproCNP:CNP ratio was lower in Enriched rats than Standard Housed rats [Main Effect of Housing: *Q*_(1, 32)_ = 12.08, *p* = 0.005]. However, an overall mean comparison was not statistically significant (Young-Standard vs. Young-Enriched *d* = 0.55 [−0.18, 1.28], *p* = 0.14). Only Young-Enriched-14-day rats had lower ratio values than Young Standard Housed rats (between group comparisons, Table 2).

**Table 2:** Between group comparisons (cohen's *d* [±95% CI]) for NTproCNP:CNP ratio within six regions of interest.

**Region**		**Young SH 14**	**Young SH 28**	**Young EE 28**	**Aged overall**
Occipital Cortex	Young EE 14 vs.	*d* = −4.34 [−6.25, −2.42]^***^	*d* = −2.28 [−3.65, −0.92]^***^	*d* = −2.0 [−3.07, −0.94]^***^	*d* = −1.33 [−2.07, −0.58]^***^
	Other Young vs.				*d* = 0.9 [0.34, 1.45]^***^
Medial Prefrontal	Young EE 14 vs.	*d* = −4.10 [−5.89, −2.30]^***^	*d* = −2.64 [−4.05, −1.23]^***^	*d* = −3.0 [−4.23, −1.76]^***^	*d* = −0.63 [−1.32, 0.05] ns
Cortex	Other Young vs.				*d* = 1.52 [0.92, 2.11]^***^
Retrosplenial Cortex	Young EE 14 vs.	*d* = −1.80 [−3.11, −0.49]^**^	*d* = −1.85 [−3.10, −0.61]^**^	*d* = −1.69 [−2.67, −0.7]^***^	*d* = −1.0 [−1.70, −0.30]^**^
	Other Young vs.				*d* = 1.24 [0.66, 1.82]^***^
Mammillary Bodies	Young EE 14 vs.	*d* = −1.85 [−1.99, −0.60]^**^	*d* = −1.59 [−2.78, −0.39]^**^	*d* = −1.20 [−2.12, −0.28]^**^	*d* = −1.21 [−1.93, −0.49]^***^
	Other Young vs.				*d* = 0.15 [−0.39, 0.68] ns
Hippocampus	Young EE 14 vs.	*d* = −0.88 [−1.99, 0.22] ns	*d* = −2.26 [−3.58, −0.93]^***^	*d* = −1.31 [−2.25, −0.38]^**^	*d* = −0.84 [−0.53, 0.15]^*^
	Other Young vs.				*d* = 0.45 [−0.08, 0.99] ns
Hypothalamus	Young EE 14 vs.	*d* = −1.82 [−3.06, −0.58]^**^	*d* = −1.43 [−2.60, −0.25]^*^	*d* = −0.68 [−1.55, 0.19] ns	*d* = −0.13 [−0.81, 0.55] ns
	Other Young vs.				*d* = 0.77 [0.21, 1.32]^**^

*EE, Enriched Environment; SH, Standard Housed; Other Young indicates pooled Young data, excluding Young-Enriched-14-day rats*.

*** *p < 0.001*;

** *p < 0.01*;

* *p < 0.05*.

NTproCNP:CNP ratio did not generally vary across groups for Aged rats (Figures 1–6). Overall, NTproCNP:CNP ratio for Aged rats was lower than Young rats (excluding Young-Enriched-14-day rats) in occipital, medial prefrontal and retrosplenial cortices, and hypothalamus (between group comparisons, Table 2), but not in mammillary bodies or dorsal hippocampus.

## Discussion

Here, we provide the first data indicating a markedly different response of CNP and its signaling system in aged compared to young rats. The major finding in Young rats, is an increased availability of CNP in all regions of interest following 14 days of enriched housing, although this effect was weak within dorsal hippocampus. This increase in CNP can be accounted for by reduced proteolytic degradation or NPR-C mediated clearance of the peptide (indicated by NTproCNP:CNP ratio reductions) as opposed to increased production. Relatively minimal modifications to peptide production (NTproCNP) were restricted to tissues from hypothalamus, retrosplenial cortex and dorsal hippocampus. In contrast, within aged rats changes to CNP concentrations were restricted to medial prefrontal cortex and related to rehousing with new cage mates with higher concentrations of CNP at 14 vs. 28 days of housing regardless of housing condition. NTproCNP:CNP ratios were reduced in comparison with young rats generally in occipital cortex, medial prefrontal cortex, retrosplenial cortex, and hypothalamus as hypothesized, but this age-related reduction was not evident in mammillary bodies or hippocampus. Compared with young rats, NTproCNP was increased in occipital cortex, and decreased in medial prefrontal cortex. Moreover, modifications to NTproCNP seen in young rats within retrosplenial cortex and dorsal hippocampus were not evident in aged rats. Varying combinations of changes to degradation and production in aged rats also resulted in increased concentrations of CNP in the aging occipital and retrosplenial cortices. Overall, these results indicate age-related modification to both the CNP signaling system broadly, and its response to environmental conditions. In addition, they indicate degradation or clearance rate to be an important factor in determining bioactivity within neural tissue.

Previous work has shown that 2 weeks of enrichment is sufficient to stimulate neural progenitor cell mobilization (Magalon et al., 2007), upregulate multiple genes associated with neuroplasticity in the hippocampus (Keyvani et al., 2004), improve cognitive performance in intact animals (e.g., Tang et al., 2001; Frick and Fernandez, 2003; Bruel-Jungerman et al., 2005) and ameliorate cognitive deficits in animals with neurological insults (e.g., Passineau et al., 2001; Hicks et al., 2002; Wagner et al., 2002). Although here we have not explicitly linked increases in CNP concentrations at this time point to other neurological or behavioral sequelae, this finding encourages the view that the peptide may contribute to such outcomes as part of the early neurological response to enrichment in young rats. Interestingly, the hippocampus is often a focus of structural and molecular responses to enrichment (see Simpson and Kelly, 2011; Hirase and Shinohara, 2014 for reviews), but here CNP exhibited the smallest effect in terms of young rats' response to short-term enriched housing. Plausibly, the size of this effect may vary throughout the hippocampal complex. Within hippocampus, CNP mRNA predominates in CA1 through CA3, whereas NPR-B mRNA is largely expressed within DG (Langub et al., 1995b; Herman et al., 1996). Substantive changes to bioactive CNP in any specific region similar to those in other regions of interest analyzed here may be masked by the inclusion of all three subregions used for hippocampal tissue analysis. This highlights a need within future studies of CNP to consider these sub-regions independently. Interestingly, an effect of enrichment on NTproCNP concentrations was seen in dorsal hippocampus for NTproCNP concentrations, suggesting a modification to gene regulation as opposed to the effect seen on bioactive CNP in other regions of interest. An explanation for this cannot be generated from this data. Future work may consider the use of PCR to analyse gene expression of Nppc directly, alongside NPR-B/NPR-C and degradative enzyme (neprilysin and insulin-degrading enzyme) gene expression to elucidate on the findings.

The relatively minimal response of aged rats to environmental manipulations used here should now encourage further study of the CNP signaling system in the aging brain. In a recent review, Mahinrad et al. (2016) outline evidence suggesting Natriuretic Peptides contribute to cognitive decline and pathologies associated with aging, which we believe this experimental work supports. Primarily, the lack of response to enrichment of bioactive CNP in aged rats compared to young is suggestive of age-related loss of sensitivity to external stimulus. Additionally, evidence of loss or change in the NTproCNP response to rehousing or enrichment in the aged retrosplenial cortex, hippocampus and hypothalamus indicates age-related modification to peptide production in these cortical nodes. Apparent accumulation of CNP with aging in occipital and retrosplenial cortices may align with the facilitation of long-term depression with aging (Kelly et al., 2006). Notably, while expected reductions to degradative activity were identified in most regions, this effect was absent in hippocampus and mammillary bodies, contrary to the hypothesis. Region specific, age-related reductions in neprilysin (the proteolytic enzyme regulating CNP) have been demonstrated previously within hippocampus (Iwata et al., 2001, 2002; Yasojima et al., 2001a,b; Higuchi et al., 2005; Hellström-Lindahl et al., 2008). Thus, the absence of hypothesized reductions to degradative activity within this region specifically raises interesting questions. Because NTproCNP:CNP ratio is influenced by both proteolytic enzyme activity and NPR-C clearance activity, one plausible line of inquiry is to examine age-related modifications to this receptor within hippocampus. Additionally, these age-related changes to production, bioactive availability and degradation or clearance of CNP should now be examined in association with other neurological factors wherein CNP may contribute to cognitive decline such as modifications to synaptic regulation, neurovascular function, anxiety, and memory (Mahinrad et al., 2016). In this context it is relevant to note that in contrast to other neuroprotective factors, the diverse molecular events potentially regulated by CNP signaling—including ion channel activity and cyclic AMP concentrations (Kuhn, 2016)—need to be considered.

The major modification to CNP in medial prefrontal cortex of aged rats seemingly related to the period of rehousing with new cage mates rather than the rehousing environment. Other minor modifications to measures from aged rats (not directly reported here, but see Figures 1, 4 Aged-Standard ratios; Figure 6 Aged-Standard NTproCNP for examples), also generally related to rehousing period. Because CNP has been previously related to anxiety regulation and related behaviors (Bíró et al., 1996; Montkowski et al., 1998; Telegdy et al., 1999, 2000; Jahn et al., 2001), this now encourages further investigation of CNP in the context of anxiety and aging, because this response in aged rats seems to relate to an anxious event (rehousing with new cage mates). CNP's effects on anxiety are dependent on corticotropin releasing hormone (Jahn et al., 2001) and medial prefrontal cortex, hippocampus and other limbic system structures participate in regulation of the hypothalamo-pituitary adrenal axis (Herman et al., 2005). Moreover, changes to hypothalamo-pituitary adrenal axis signaling are thought to contribute to neurological and behavioral changes during aging (Mizoguchi et al., 2009; Swaab and Bao, 2011) as are molecular and physiological changes throughout these same frontal and temporal cortical networks (Nestor et al., 2003). Although anxious and mnemonic behaviors were not studied here, age-related modifications to this peptide system identified within fronto-temporal regions provides initial experimental support for an association with cognitive decline (Mahinrad et al., 2016). However, a lack of reported impairment in cognition in the homozygous human loss-of-function mutation of the NPR-B receptor (Wang et al., 2016) suggests at least two additional caveats. First, is that this conclusion may be limited to rodents. Cognitive and neurological effects have been reported in rodents with a loss of NPR-B function (Tamura et al., 2004; Barmashenko et al., 2014; Buttgereit et al., 2016). Alternatively, it may be that the NPR-C receptor is of greater importance in mediating central functions of CNP than is currently believed, but suggested by others (Trachte, 2000, 2005; Gong et al., 2017; Moghtadaei et al., 2017). Clearly further work is required to clarify the importance of CNP and its receptors in regulating neural function in both rodents and humans. As the first report of CNP and related molecules within rodent medial prefrontal cortex and retrosplenial cortex, and changes to bioactive CNP during environmental stimulation, the current findings now call for more focused studies of CNP activity, function and receptor expression throughout these important neural circuits.

## Data availability statement

Data generated and analyzed in this experiment is available from corresponding author on request.

## Author contributions

JD-A and EE developed initial concept for young rats, which was expanded to aged rats in consultation with SR and TP. SR conducted animal trials, tissue acquisition and homogenization, peptide assays for aged rats, data analysis, and visualization. TP conducted assays for young rats and supervised aged rat assays. SR prepared the manuscript in consultation with all other co-authors.

### Conflict of interest statement

EE is a consultant for BioMarin pharmaceutical. The remaining authors declare that the research was conducted in the absence of any commercial or financial relationships that could be construed as a potential conflict of interest.
